# Comment on "Encapsulating Peritoneal Sclerosis in a kidney transplant recipient: Case Report"

**DOI:** 10.1590/2175-8239-JBN-2020-0253

**Published:** 2021-04-19

**Authors:** Gioacchino Li Cavoli, Rosalia Mongiovi, Barbara Oliva, Antonio Amato, Angelo Tralongo

**Affiliations:** 1 Civico Hospital Palermo Italy, Nephrology Dialysis Renal Transplantation Departament, Palermo Italy.

**Dear Editor**

We read with interest the article by Ribeiro et al. (2020)^1^, about the Encapsulating Peritoneal Sclerosis (EPS) in subjects on peritoneal dialysis (PD). When exposed to PD dialysis solutions, the peritoneal membrane undergoes some morphological changes. Most patients develop the Simple Peritoneal Sclerosis in which a thin layer of submesothelial fibrosis is often apparent, with a thickness not exceeding a few hundred microns, and a component of neoangiogenesis, without significant vascular damage, is often demonstrable. Calcifications are rare and so are the signs of a significant inflammatory state. A minority of patients on PD develops the EPS, a rare complication of long-term PD, which consists in a progressive inflammatory process involving both visceral and parietal peritoneum, leading to encapsulation of the adhered intestinal tract. EPS features marked fibrosis, acute and chronic inflammation, widespread calcification, and vascular thickening. Some authors think that Simple Peritoneal Sclerosis and EPS are the extremes of the continuous spectrum of a single disease related to PD biocompatibility. EPS may become clinically apparent when patients are on PD (classical EPS) or after undergoing kidney transplantation (post-transplantation EPS). This presentation of EPS seems to occur shortly after kidney transplantation in former PD patients. The critical phase for post-transplantation EPS is during the first year after transplantation^2^.

Our experience: a 61-year-old patient, for 10 years on PD, underwent kidney transplantation from deceased kidney donors. A few days afterwards, she started to complain about dyspeptic digestive symptoms. The peritoneal catheter was removed and biopsy of peritoneal membrane was carried out. The peritoneal histological examination showed signs of peritoneal sclerosis: thickening of peritoneal membrane (> 600 micrometers), progressive fibrosis from the submesothelial layer towards the inside layer, and marked thickening of middle vascular wall and mesothelial denudation. We did not find signs of active or chronic inflammation or peritoneal calcifications. The Abdomen Contrast-Computed Tomography (CT) showed adherent and conglomerate intestinal loops (cocooning) (Figure 1). Based on symptomatology and radiological and histological findings, we diagnosed a recent onset of EPS. Due to the profibrotic effects of calcineurin inhibitors, we stopped treatment with tacrolimus. Because of the evidence supporting protection against the development of EPS exerted by inhibitors of the mammalian target of rapamycin, we started therapy with everolimus^3^^,^ 4. Besides a transient increase in steroid therapy, we started therapy with tamoxifen^5^. In the following 6 months of follow-up, the patient's symptoms ameliorated and her condition improved with regard to the bowel sub-occlusive crisis. The risk of EPS increases with longer time on PD. Probably unidentified factors make some patients more susceptible to developing EPS.

**Figure 1:**
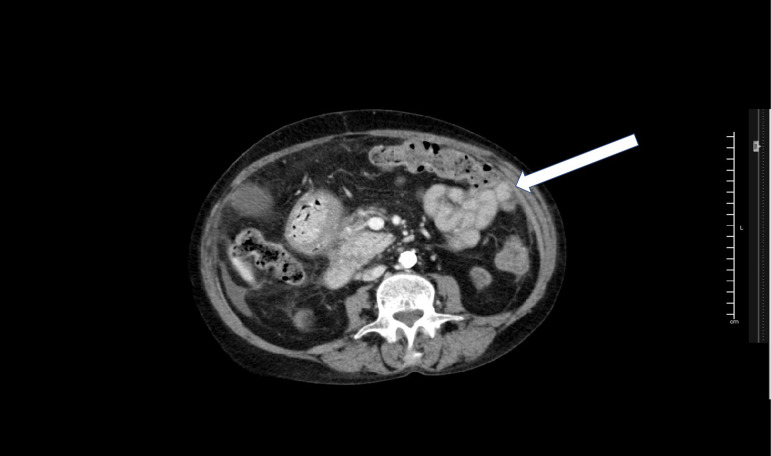
Abdomen contrast-CT showing adherent and conglomerate intestinal loops (white arrow).

## References

[1] Ribeiro BHD, Takenaka VS, Borges FS, Andrade TF, Lessa SB, Mancero JMP (2020). Encapsulating peritoneal sclerosis in a kidney transplant
recipient - case report. Braz J Nefrol.

[2] Garosi G, Mancianti N, Corciulo R, La Milia V, Virga G (2013). Encapsulating peritoneal sclerosis. J Nephrol.

[3] Huddam B, Azak A, Koçak G, Başaran M, Voyvoda N, Duranay M (2012). Additive effectiveness of everolimus plus tamoxifen therapy in
treatment of encapsulating peritoneal sclerosis. Ren Fail.

[4] Ghadimi M, Dashti-Khavidaki S, Khalili H (2016). mTOR inhibitors for management of encapsulating peritoneal
sclerosis: a review of literatures. Ren Fail.

[5] Mohamed AO, Kamar N, Nogier MB, Esposito L, Duffas JP, Rostaing L (2009). Tamoxifen therapy in kidney-transplant patients presenting with
severe encapsulating peritoneal sclerosis after treatment for acute humoral
rejection. Exp Clin Transplant.

